# Improving the reporting and sound methodology of scientific articles: introduction of an editorial series

**DOI:** 10.2340/17453674.2026.46155

**Published:** 2026-06-04

**Authors:** Søren Overgaard, Robin Christensen, Cecilia Rogmark, Jeppe Vejlgaard Rasmussen, Ivan Hvid, Marianne Westberg, Ilkka Helenius, Keijo Mäkelä, Taco Gosens, Per-Henrik Randsborg, Bart G Pijls, Paul Gerdhem, Philippe Wagner, Li Felländer-Tsai, Aleksi Reito

**Affiliations:** 1 Department of Orthopaedic Surgery and Traumatology, Copenhagen University Hospital Bispebjerg Frederiksberg, Denmark;; 2 Department of Clinical Medicine, University of Copenhagen, Denmark; 3 Section for Biostatistics and Evidence-Based Research, the Parker Institute, Bispebjerg and Frederiksberg Hospital, Copenhagen, Denmark; 4 Research Unit of Rheumatology, Department of Clinical Research, University of Southern Denmark, Odense University Hospital, Denmark;; 5 Faculty of Medicine, Lund University, Lund, Sweden; 6 Department of Orthopedics, Skåne University Hospital, Malmö, Sweden; 7 Department of Orthopaedic Surgery, Herlev and Gentofte University Hospital, Denmark; 8 Division of Orthopaedic Surgery, Oslo University Hospital, Oslo, Norway; 9 Department of Orthopaedics and Traumatology, University of Helsinki and Helsinki University Hospital, Helsinki, Finland; 10 Elisabeth Tweesteden Hospital, Tilburg, the Netherlands; 11 Department of Medical and Clinical Psychology, Tilburg University, Tilburg, the Netherlands; 12 Department of Orthopedic Surgery, Akershus UniversityHospital, Norway; 13 Institute of Clinical Medicine, Campus Ahus, Faculty of Medicine, University of Oslo, Norway;; 14 Department of Orthopaedics, Leiden University Medical Center, Leiden, The Netherlands; 15 Dutch Arthroplasty Register (Landelijke Registratie Orthopedische Interventies), ‘s-Hertogenbosch, The Netherlands; 16 Department of Orthopaedics and Traumatology, Turku University Hospital, and University of Turku, Turku, Finland; 17 Department of Surgical Sciences, Orthopaedics and Hand Surgery, Uppsala University, Department Orthopaedics, Sweden; 18 Centre for Clinical Research, Uppsala University, Region Västmanland, Västerås, Sweden; 19 Division och Orthopedics and Biotechnology, Department of Clinical Science, Intervention and Technology (CLINTEC), Karolinska Institutet, Stockholm, Sweden; 20 Trauma, Emergency Surgery and Orthopedics, Karolinska University Hospital, Stockholm, Sweden; 21 Department of Orthopaedics and Trauma, Tampere University Hospital, Tampere, Finland

**Why is it important to improve reporting?**

- Ensure content and structure
- Transparency
- Appropriate presentation
- Quality improvement
- Replication of your study
- Proper communication
- Requirement for interpretation of methodological quality
- Unbiased evidence
- Reduce the number of review rounds
- Getting your paper published
- Improves inclusion in systematic reviews and meta-analyses

The editorial team is pleased to introduce a new series of editorials, focusing on improving the reporting and sound methodology of scientific articles.

This initiative is not intended to replace established reporting guidelines, such as CONSORT and STROBE, or any other guidelines, all of which are available from the Equator Network. The aim is to complement them by drawing attention to common weaknesses in submitted manuscripts. We also aim to provide guidance on how to improve clarity, transparency, and precision.

Incomplete or unclear reporting limits the ability of editors and reviewers to assess a manuscript. If unchanged, it will also affect the reader’s ability to assess the validity and relevance of the published work. We will highlight topics that we believe will further enhance the quality of your manuscript, to make the research interpretable and reproducible.

*Acta Orthopaedica* has a continuing focus on this topic, as we have recently published articles addressing Guidelines for a structured manuscript, as well as others on methodological and statistical considerations [1-4]. These standards are intended to ensure that submitted manuscripts can be evaluated efficiently without too many reviews. This will reduce the time spent by the authors and the editors, resulting in faster publication.

We believe that authors benefit from clear guidelines. This is why it is important to follow the reporting guidelines. They play a crucial role in ensuring transparency and consistency. *Acta Orthopaedica* expects authors to respect the appropriate standards as outlined in our submission requirements. *Acta Orthopaedica* supports the ICMJE’s (International Committee of Medical Journal Editors) policy on recommendations for the Conduct, Reporting, Editing, and Publication of Scholarly Work. *Acta Orthopaedica* also supports the development and adherence to field-specific reporting guidelines. The recently updated guideline for radiostereometric analysis studies is an excellent example of this [5].

The reporting guidelines define a checklist that, as a minimum, should be followed in a manuscript. Adhering to these guidelines helps the authors to present their work in a clear, accurate, and systematic manner, making it easier for reviewers and readers to understand. In addition, it increases transparency and supports reproducibility. Improved reporting also increases the likelihood of inclusion in systematic reviews and meta-analyses, which is important for the evidence base for a particular topic. While the guidelines ensure the content of the study, they do not stipulate how you should communicate in every aspect. Once the study has been conducted, the role of the manuscript is to communicate what was planned and done, and what was found—without post hoc reinterpretation, selective emphasis, or speculative framing. Creative or data-driven reporting risks introducing spin and undermining the credibility of the findings. Reporting of results should be structured, transparent, and closely aligned with the original objectives, enabling readers to assess the validity and relevance of the work without ambiguity.

*Acta Orthopaedica’s* new series of editorials will focus on specific topics important for proper scientific reporting. This includes every part of the manuscript from the title to the conclusion, and how references are used to support statements. This could be design-specific guidance on how manuscripts should be structured, including preferred outlines and the presentation of key figures and tables to support clear and consistent reporting.

Although a peer-review process may change a manuscript considerably, a well-prepared manuscript at submission increases the likelihood that the manuscript will go into review without too many review rounds, and eventually be accepted.

Ultimately, improved reporting and sound methodology of published articles will ensure that regulatory and public health decision-making is based on evidence with reduced risk of bias.
